# Structural Changes in Resin-Based Composites in Saliva of Patients with Leukemia before Starting Chemotherapeutic Regimen

**DOI:** 10.3390/polym14030569

**Published:** 2022-01-31

**Authors:** Alexandru Mester, Marioara Moldovan, Stanca Cuc, Ioan Petean, Ciprian Tomuleasa, Andra Piciu, Cristian Dinu, Simion Bran, Florin Onisor

**Affiliations:** 1 Department of Oral Health, University of Medicine and Pharmacy “Iuliu Hatieganu”, 400012 Cluj-Napoca, Romania; mester.alexandru@umfcluj.ro; 2 Department of Polymer Composites, Institute of Chemistry “Raluca Ripan”, University Babes-Bolyai, 400294 Cluj-Napoca, Romania; 3 Faculty of Chemistry and Chemical Engineering, University Babes-Bolyai, 400294 Cluj-Napoca, Romania; ioan.petean@ubbcluj.ro; 4 Department of Hematology, Institute of Oncology “Ion Chiricuta”, University of Medicine and Pharmacy “Iuliu Hatieganu”, 400012 Cluj-Napoca, Romania; ciprian.tomuleasa@umfcluj.ro; 5 Department of Medical Oncology, Institute of Oncology “Ion Chiricuta”, University of Medicine and Pharmacy “Iuliu Hatieganu”, 400012 Cluj-Napoca, Romania; andra.piciu@umfcluj.ro; 6 Department of Maxillofacial Surgery and Implantology, University of Medicine and Pharmacy “Iuliu Hatieganu”, 400012 Cluj-Napoca, Romania; dr_brans@umfcluj.ro (S.B.); florin.onisor@umfcluj.ro (F.O.)

**Keywords:** leukemia, dental composite, chemotherapy, saliva, oral health

## Abstract

**Background:** The aim of this in vitro study was to assess the morphological characteristics and stability of dental composites immersed in saliva collected from patients with leukemia. **Material and Methods:** A total number of five patients without systemic disease and 20 patients with leukemia (acute lymphoblastic leukemia (ALL), acute myeloid leukemia (AML), chronic lymphocytic leukemia (CLL), and chronic myeloid leukemia (CML)) were included for saliva sampling. Composite disks were immersed in the leukemia, control, and artificial environments for 7 days. At the end of the experiment, atomic force microscopy (AFM), color stability (ΔE), and saliva elements analysis were performed. Statistical significance was considered for a *p*-value under 0.05. **Results:** The most changed surface resulted for ALL with a roughness that was almost double that of the untreated sample and was significantly increased compared to the healthy saliva. The effect of CLL was not as intense as observed for acute leukemia, but was significantly over the control. ALL seemed to modify structural components of the saliva, which were able to deteriorate the surface of the composite. ALL saliva promoted a significant dissolution of the initial feature of the samples and promoted nano-particle clusterization. All dental composites showed clinically acceptable color change values (ΔE < 3.3) in all four-leukemia salivas; CLL and CML showed large color differences for all composites. The total concentrations of P, Na, and K showed wide ranges of variations, while the coefficient of variation in Fe, Cu, and Mg showed narrow variations between the salvias’ investigated. The salivary concentration of zinc decreased considerably in the CLL and CML environments compared to the ALL and AML environments. Fe and Cu were significantly increased in the CML environment. **Conclusions:** Control and artificial salivas have a mild erosive effect on the surface of dental composites. The acute stage of the disease seems to deteriorate the surface roughness rather than its morphology, however, in the chronic stage, it is the surface morphology that mostly deteriorates.

## 1. Introduction

Leukemia represents a well-known malignant disease of uncontrolled proliferation of white and red blood cells [1,2]. Due to the impairment of the blood cells and the presence of immunosuppression, patients present with several oral complications such as cervical lymphadenopathy, petechiae, oral ulcers, and mucositis [1,2]. Gingival bleeding is another common oral sign in patients with leukemia and some reports have stated that it is the initial oral sign in 17.7% of acute leukemia patients and 4.4% in patients with chronic leukemia [3]. Gingival enlargement has also been reported; initially, beginning in the interdental papilla and then evolving to the marginal and attached gingiva [2]. Gingival bleeding in association with gingival enlargement and lack of dental care leads to plaque and calculus accumulation and inflammation, which enable the initiation of oral infection; it is worth mentioning that up to 25–54% of septic episodes have an oral infection source [4]. 

Besides oral mucosa and periodontal infections, leukemia patients are subjected to tooth caries [5,6]. Angst and coworkers published a meta-analysis in which the pooled decayed, missing, and filled tooth (DMFT) index means were 2.28 and 3.65 for patients before and after chemotherapy for leukemia [5]. Hong and coworkers found that the prevalence of dental caries in patients who received chemotherapy was 37.3% [7]. Teeth caries may develop due to high doses of chemotherapy, which lower pH value and buffer the ability of saliva; severe pancytopenia and a suppressed immune system are other factors that determine an increased presence of dental caries [6,7,8]. Another research paper published by Hansen and coworkers indicated that a conservative dental approach resulted in low odontogenic complications in patients before starting hematopoietic stem cell transplantation (HSCT) [9].

Oral complications (e.g., deep dental caries) require immediate dental treatment in order to prevent a life-threating situation in patients who are about to receive a hematopoietic stem cell transplant [1,3,4]. When it comes to dental caries, teeth should be treated with the minimum involvement of the pulp. Cancer patients are mostly treated using an amalgam, composite, or glass ionomer restoration before/during/after chemotherapy [4]. The evidence published by Hong suggests that conventional glass ionomer restorations performed more poorly than resin-modified glass ionomers, composite resins, and amalgam restorations in patients with head and neck radiation [7,8]. It is well stated that odontogenic infections are mostly related to improper dental treatment, rather than the potential myelosuppression determined by chemotherapy [10]. The oncology team should pay attention to dental infections due to the fact that these disorders may change the course of the chemotherapeutic regimen [11]. Other researchers found that DMFT scores increased post HSCT [12], and found an association between dental decays and HLA types [13]. In regards to this, it is mandatory that dentists and oncologists/hematologists establish an interdisciplinary collaboration. All leukemia patients should undergo a detailed oral examination and dental radiology in order to assess potential oral foci, treat possible oral disorders, and keep a healthy oral status during chemotherapeutic regimens [14,15]. 

It is well known that patients who are receiving hematopoietic stem cell transplant are systematically compromised, and thus a good oral hygiene cannot be maintained [1,4]. In order to prevent the initiation of dental caries, appropriate dental restorative material is needed. So far, there are no guidelines mentioning which dental composite should be used in leukemia patients. In regards to the last statement, the aim of our in vitro study was to investigate the effects of leukemia salivary contamination before chemotherapy on four dental composites. 

Many etiological aspects of degradation of dental composite in both artificial and human salivary environments have been evaluated [16,17,18], but the differences between the four types of leukemia, before the start chemotherapy regimens, have not been investigated. The role of saliva in maintaining oral function is a constant factor to consider as a preventive effect in the subsequent occurrence of leukemia disease complications. Factors such as differences in salivary characteristics that result in a different composition of elements [19], may lead to a change in the aesthetics and surface morphology of dental composites and, implicitly, in the patient’s quality of life. Therefore, the aim of our in vitro study was to assess the morphological characteristics and stability of dental composites immersed in saliva collected from patients with leukemia. 

## 2. Material and Methods

### 2.1. Preparation Saliva Sampling and Dental Composite Systems

Before starting out our in vitro research, an IRB approval (183/25.06.2020) was obtained from the ethical board of Institute of Oncology “Prof. Dr. Ion Chiricuta”, Cluj-Napoca, Romania. A total number of 5 patients without systemic disease and 20 patients with leukemia (acute lymphoblastic leukemia (ALL), acute myeloid leukemia (AML), chronic lymphocytic leukemia (CLL), and chronic myeloid leukemia (CML)) were included. All included patients agreed with the study protocol and signed the informed consent. The inclusion criteria for our patients were: no gingivitis/periodontitis, at least 20 teeth present, no fillings, and no use of alcohol or tobacco. Saliva sampling was performed according to the protocol of Berge et al. [20]. Four nanohybrid composite resins used for both anterior and posterior fillings (G-aenial Anterior A2, GC corporation, Japan; Herculite XRV Ultra A2, Kerr, Italy; Evetric A2, Ivoclar Vivadent, Liechtenstein; Enamel Plus HRi, UD2, Micerium, Italy) (Table 1) were investigated using the artificial saliva and in saliva collected from patients with/without leukemia. For each composite, 18 disc-shaped specimens (1 mm thickness × 10 mm diameter) were prepared using a Teflon mold matrix. Specimens were polymerized in 5 points for 20 s/point using a photopolymerizable lamp Led.B (Woodpecker) with an intensity of 1000 mW/cm^2^. The materials were initially investigated, i.e., immediately after the polymerization process and after 7 days of immersion in different storage media (ALL, AML, CLL, CML, Artificial, Control). Saliva was collected and changed daily and the body temperature was simulated by immersing the saliva samples in a laboratory water bath at a temperature of 37 °C.

### 2.2. Atomic Force Microscopy and Scanning Electron Microscopy Analysis

The dental composites discs have plain parallel surfaces being suitable for the Atomic Force Microscopy (AFM) investigation. The samples exposed to saliva were extracted from the storage recipient and washed with bi-distilled water to remove any traces, and then dried in a desiccator. The completely dry state of the discs is an AFM requirement. The AFM investigation was effectuated with a JEOL JSPM 4210 Scanning Probe Microscope, Tokyo, Japan, operated in AC mode using NSC 15 cantilevers (MikroMasch Co, Bulgaria, Sofia). The cantilevers have a resonant frequency of 325 kHz and a force constant 40 N/m. Each sample was scanned in at least five different macroscopic areas where the topographic images were recorded in an area of 5 × 5 µm. The images were processed according to the standard procedures using JEOL Win SPM 2.0 processing software following 2D and 3D representation of the topographic images, and measuring of the surface roughness was expressed as Ra and Rq. Both the roughness parameters are important to illustrate the surface changes induced by saliva. Ra is described as an arithmetical mean deviation in the surface height of each pixel from the topographic image, and Rq represents the root mean squared deviation in the surface height of each pixel from the topographic image. Ra and Rq are measured for the entire topographical image, also known as area roughness, and the values are calculated by the processing software. 

Scanning electron microscopy (SEM) was used to investigate the structure of the dental composites disc investigated for the AFM. The investigation of the samples was performed using INSPECT S electron microscope of the FEI company (Hillsboro, OR, USA), at low vacuum, with an acceleration voltage of 30 kV. SEM images were captured initially and after 7 days of immersion in the study’s salivas (magnification 5000×).

### 2.3. Color Stability

Color stability was measured with spot measurement devices, which measure a small area on the composite surface using Vita Easy Shade Advance 4.0. system. According to the CIE (Commission Internationale de l’Eclairage) L*a*b* system, CIE-L*a*b* values are measured relative to a standard illuminant (A) against a white background, with L* representing lightness (white–black axis) and a* and b* representing chroma (green-red and blue-yellow, respectively) [21]. Each specimen was measured three times, and the mean values for L*, a*, and b* were recorded, thus obtaining the initial values. Then, the specimens were divided into 6 subgroups, which were immersed in different environments: ALL, AML, CLL, CML, Artificial saliva, and Control. Color measurements were obtained again after 7 days storage at 37 ± 1°C, and color change (Δ*E*) was calculated using the following formula:

$$\Delta E={\left[{\left(L_{1}^{*}-L_{0}^{*}\right)}^{2}+{\left(a_{1}^{*}-a_{0}^{*}\right)}^{2}+{\left(b_{1}^{*}-b_{0}^{*}\right)}^{2}\right]}^{1/2}$$

### 2.4. Saliva Elements Analysis

Trace elements were determined using an inductively coupled plasma optical emission spectroscopy-Optima 2100 (ICP-OES, PerkinElmer, Shelton, CT, USA). This is a highly sensitive technique that allows the determination of many elements in volumes of minimal biological samples [22]. Thus, the purpose was to assess the relationship between the levels of the elements of the salivary state in patients with different leukemia types compared to batches of healthy and artificial controls. In ICP-OES, samples were introduced as dilute solutions of frozen saliva and freeze-dried after the 7-day immersion of the dental composites for different elements (Zn, Ca, P, Fe, Cu, K, Mg, Na) present in the saliva composition.

### 2.5. Statistical Analysis

For color determination (ΔE), each sample was tested at 3 different points. The obtained results were considered for statistical analysis in order to compare the storage medium variables. Thus, the statistical difference between the groups was evaluated using the ANOVA One-Way test, and for post-hoc comparisons between groups, the Tukey test, the level of significance being set at α = 0.05. The statistical analysis was performed using the Origin2019b Graphing and Analysis graph. Statistical significance was achieved when *p* value was 0.05. 

## 3. Results

### 3.1. AFM and SEM Analysis

**Figure 1:**
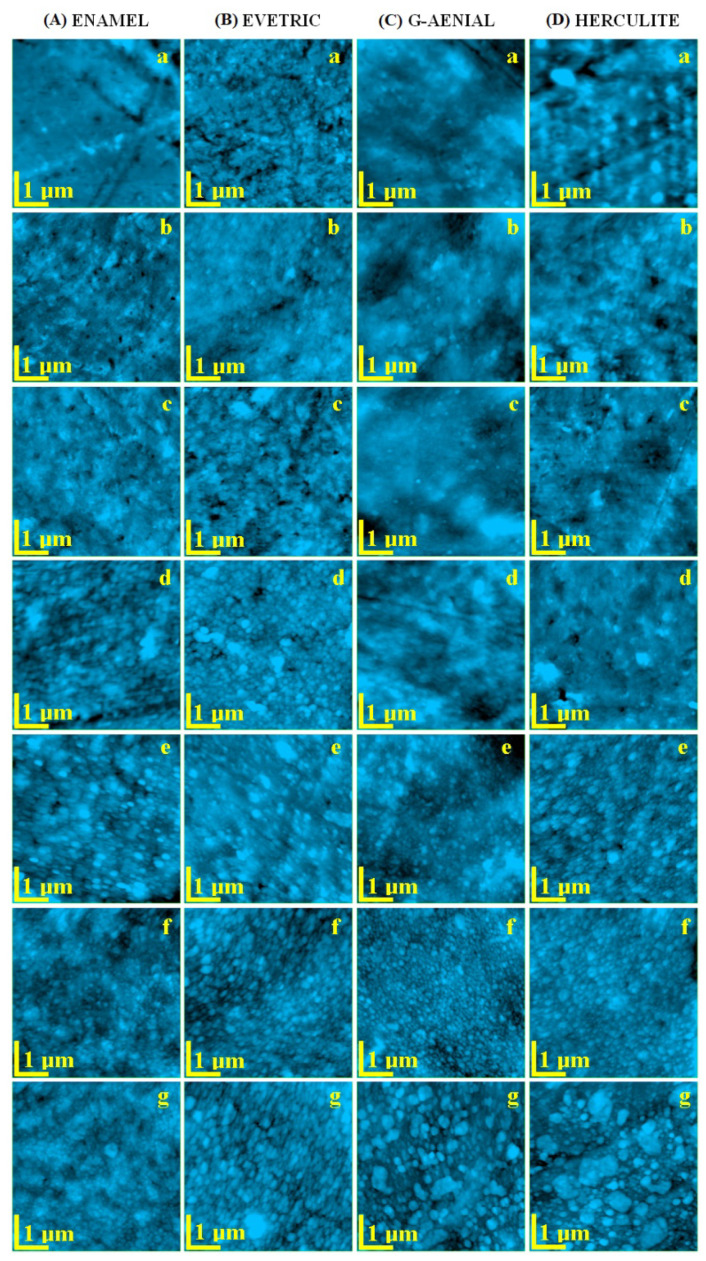
AFM topographical images for dental composites (**A**) Enamel, (**B**) Evetric, (**C**) G-aenial, and (**D**) Herculite exposed to various saliva: (**a**) untreated sample, (**b**) healthy, (**c**) artificial, (**d**) AML, (**e**) CML, (**f**) ALL, (**g**) CLL.

The fine microstructure evolution of the dental composites was investigated with AFM to observe the changes that occurred to the surface morphology and the roughness caused by exposure to saliva (Figure 1). The effect of leukemia was followed by a complex system of reference samples: untreated materials (Figure 1a), materials exposed to the healthy saliva (Figure 1b), and materials exposed to artificial saliva (Figure 1c). Therefore, the effect induced by the saliva collected from patients with leukemia will be evidenced compared to the healthy saliva, in addition to the effect of healthy saliva on the untreated materials.

**Figure 2:**
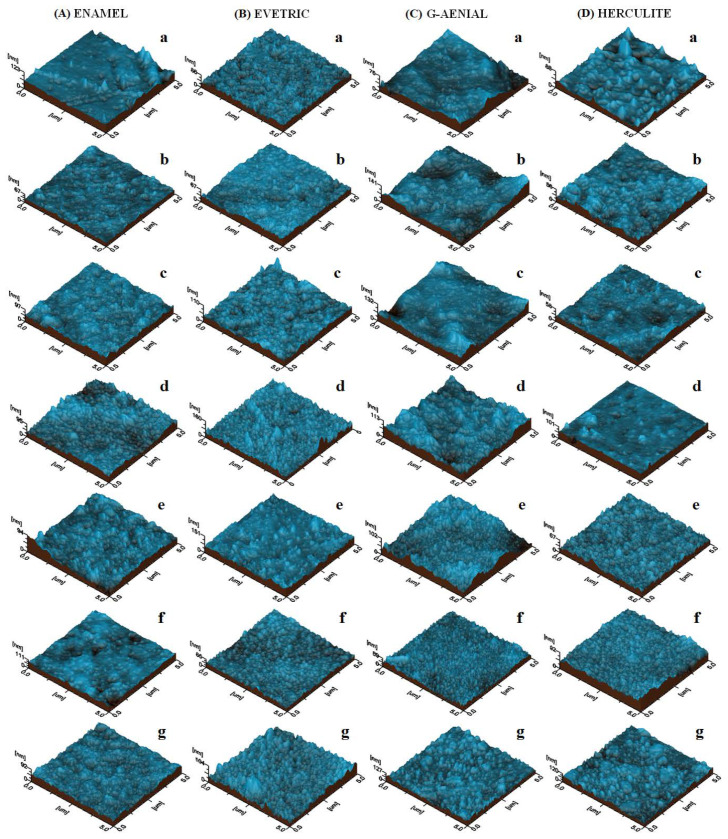
AFM 3D topographical images for dental materials: (**A**) Enamel, (**B**) Evetric, (**C**) G-aenial, and (**D**) Herculite exposed to various saliva: (**a**) untreated sample, (**b**) healthy, (**c**) artificial, (**d**) AML, (**e**) CML, (**f**) ALL, (**g**) CLL.

Enamel is a dental composite based on a nano-structured filler well dispersed onto the polymer matrix. Therefore, the initial sample features a very compact morphology proving an optimal cohesion between the polymer and the filler nano-particles, which leads to a relative smooth topography (Figure 1Aa). Some of the sample handling traces occur on the left side of the image, but do not influence the morphological aspect of the composite, as observed in the 3D image in Figure 2Aa. Surface roughness is Ra = 5.28 nm and Rq = 7.42 nm, proving the smoothness of the Enamel composite.

The exposure to healthy and artificial saliva deteriorates the Enamel composite surface in a similar manner. The polymeric outermost layer interacts with the wet environment in saliva, which is slightly eroded, revealing the internal structure of the composite. The filler nano-particles are well dispersed and embedded into the matrix (their diameter capped with polymer is about 80 nm). Some clustering tendency occurs among the nano-particles. The clusters are formed by the coalescence of rounded nano-particles in submicron domains that have an irregular shape. Their size varies from about 200 to 500 nm. This is very well observed on the surface topography after exposure to the healthy saliva (Figure 1Ab), and the clustering is more evident after exposure to the artificial saliva (Figure 1Ac). The situation leads to an increasing roughness of the exposed samples; the values obtained for artificial saliva are slightly increased compared to the healthy saliva (Table 1).

Myeloid leukemia has influenced the saliva composition and is expected to change the Enamel composite surface (Figure 1Ad,Af). The erosion after exposure was more intense than the one observed for healthy saliva and seems to be more intense for acute leukemia. This finding is sustained by the roughness variation of 8.78 nm for AML and 6.24 nm for CML. The filler clusters are modified by some components of myeloid leukemia saliva; they become more rounded and their diameter increases in a range of about 300–800 nm for AML (Figure 1Ad) and 400–800 nm for CML (Figure 1Ae). The tridimensional aspect of cluster modification is observed in Figure 2Ad,Af. The AFM data show that AML saliva deteriorates the Enamel composite in a more accentuated manner than CML. Following the Tukey test, for Ra and Rq, significant differences were found between AML and other types of saliva.

Evetric is also a composite based on a polymer matrix and a nano-structured filler. The AFM investigation of the untreated sample reveals a uniform surface with nano-particles well dispersed into the polymer bulk having diameters of about 60 nm (capped with polymer) and some small clusters of about 150–200 nm (Figure 1Ba). The cluster presence in the untreated surface leads to a significant roughness of Ra = 6.82 nm and Rq = 9.08 (Table 2). 

The exposure to the healthy saliva had a mild effect on the Evetric surface (Figure 1Bb) consisting of some erosion, which slightly increases the surface roughness and gives a washed-up aspect to the microstructure. Besides this, the artificial saliva has a more intense effect on the Evetric surface. Micro- and nano-structural changes occur (Figure 1Bc). The observed number of clusters increases, and their diameter grows to be about 100 nm over the values observed in the untreated state (some of them are around 500 nm, upper side of Figure 1Bc). This situation shows that the Evetric composite is sensitive to some components in the artificial saliva.

Acute myeloid leukemia is expected to deteriorate the surface of the Evetric composite. AML (Figure 1Bd) leads to a significant increase in the cluster number and diameter to a range of 200–400 nm. The effect is more intense than the one observed on the artificial saliva leading to a roughness boost (Table 2). The exposure to the CML saliva modifies the surface in a similar manner but not quite identical. The number of clusters increases slowly but their diameter is significantly bigger with some of them being situated at around 800 nm (Figure 1Be) and are surrounded by washed areas, which indicate mineral loss. Therefore, the roughness increasing is not as high as compared to the acute stage. Both stages, the acute and chronic forms, prove to significantly deteriorate the surface of the Evetric composite because of some components of the saliva that do not occur in healthy saliva. Following the Tukey test, for Ra and Rq, significant differences were found between AML and the unexposed and healthy types of saliva.

G-aenial composite is a complex mixture containing a polymer matrix and a complex filler system based on 16 nm silica nano-particles; 100 nm lanthanoid fluoride; 400 nm strontium glass; and 850 nm silica glass. It leads to a complex surface, which was investigated by AFM. The polymer matrix tends to form a smooth surface and the complex filler system is dispersed randomly in the material bulk. Some of the components are close enough to the surface to be seen in Figure 1Ca. The finest nano-particles are so well dispersed and embedded in the polymer that they are not visible in the topographic image, however, submicron filler particles such as lanthanoid fluoride, strontium glass, and silica glass are observed on a nearby surface, but are covered with a thin layer of polymer.

The exposure to healthy and artificial saliva apparently does not deteriorate the G-aenial topography (Figure 1Cb,Cc). The morphology is almost unchanged but the surface roughness is significantly increased. This finding could be explained by a partial washing of some of the polymer layer without mineral loss or filler shape changes. 

Saliva collected from AML determined severe morphological changes to the G-aenial composite sample. The smooth polymer surface is deeply eroded, which brings the filler particles to the top. It seems that lanthanoid fluoride nano-particles (initially about 100 nm diameter) react with some of the saliva components and become bigger on the surface having diameters of about 300 nm (Figure 1Cd). This finding is more visible in a tri-dimensional view (Figure 2Cd). Therefore, the roughness presents a strong increase compared to the initial material, but slightly lower than the one observed for healthy saliva. Similar behavior is observed for CML, but the erosion presents more drastic changes to the surface topography, the erosion seems to change even the smaller nano-particles, which tend to form clusters of about 70–80 nm on the surface (Figure 1Ce). The roughness measured for CML is comparable with the one observed for AML. Following the Tukey test, significant differences were found between unexposed G-aenial samples and those immersed in AML, CML, and CLL. Statistical differences were also found between the sample surfaces exposed in artificial and healthy saliva.

Herculite is a nano-hybrid composite based on nano-particle filler designed to assure optimal workability associated with the desired degree of surface polishing. The AFM investigation of the initial sample reveals an irregular surface with some clusters in the range of 300–800 nm in diameter very well coated with polymer (Figure 1Da). Despite the irregular morphology, the roughness of the initial sample is comparable to the other composites tested in present article. This is due to the outermost layers of polymer that cover the nano-structural asperities.

The exposure to the healthy and artificial saliva is expected to remove the outermost layers of polymer and to expose the observed submicron formations. Despite all expectations, both cases lead to a smooth and compact surface with nano-particles of about 60 nm well embedded into the matrix and only few nano-clusters of about 120–180 nm in diameter (Figure 1Db,Dc). This leads to a significant decrease in the roughness compared to the initial state. The nano-structural evolution leads to the dissolution of the surface irregularities (caused by the molding process) at the area of saliva exposure, which reveals the uniform and smooth consistence of the composite bulk. 

The AML saliva acts very similarly to the healthy saliva as well as to that of the surface topography and roughness (Figure 1Dd). The morphology changes occur on the surface after exposure to the CML saliva. The profound dissolution of the upper layers of the Herculite composite sample leads to the exposure of the nano-particle structure to the medication residues from the saliva, which causes their clusterization (Figure 1De). The nano-particles changed by erosion tend to regroup into clusters having about 200 nm with a washed-up aspect. This leads to a significant decrease in the roughness compared to the initial state and to the healthy saliva. Following the Tukey test, for Ra and Rq, significant differences were found between samples immersed in CLL and all other immersion media.

**Figure 3:**
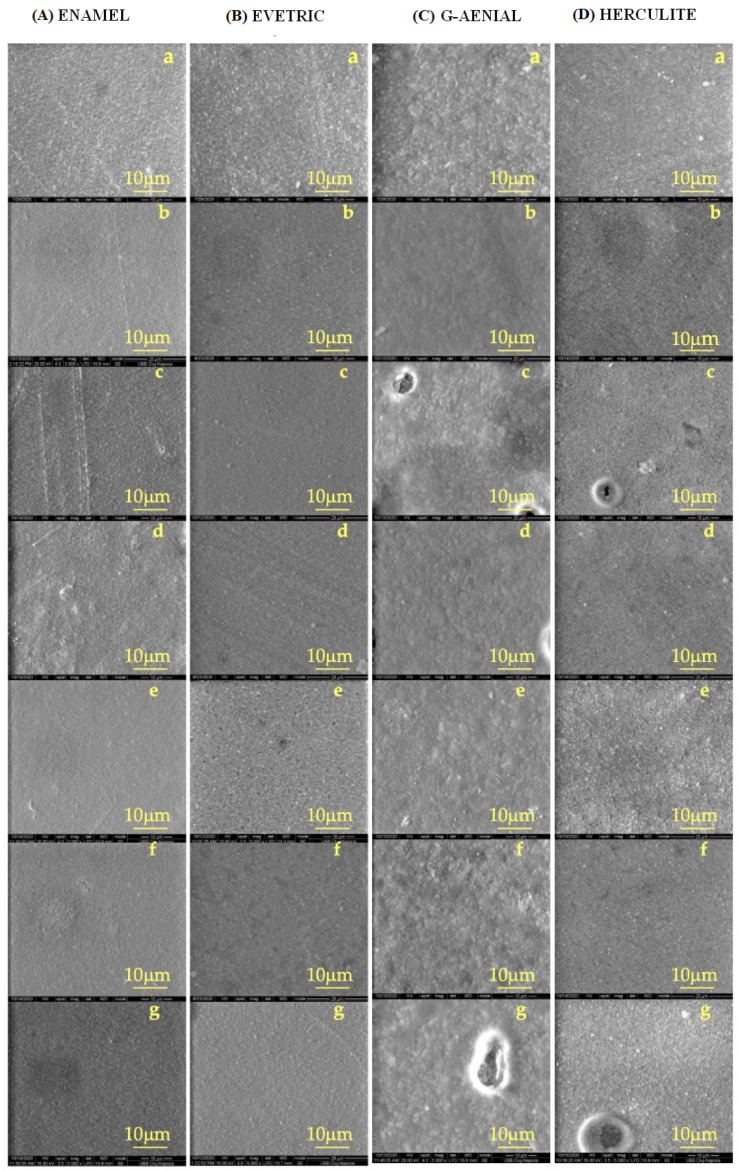
SEM images for dental composites: (**A**) Enamel, (**B**) Evetric, (**C**) G-aenial, and (**D**) Herculite exposed to various saliva: (**a**) untreated sample, (**b**) healthy, (**c**) artificial, (**d**) AML, (**e**) CML, (**f**) ALL, (**g**) CLL.

Following the SEM images (Figure 3), it can be seen that after the 7 days of immersion in healthy saliva, the composites kept the same uniform surface (Figure 3B), just like the untreated samples (Figure 3A). Different immersion environments on the surface of the materials indicate deformations and fractures following the process of degradation. For the G-aenial composites, the surfaces underwent the most visible changes compared to the initial and control group (Figure 3A,B), causing gaps in CLL and artificial saliva. In the same immersion environments, gaps were found for the Herculite composite. The enamel composite material is the one that indicates less erosion on surfaces after being in contact with saliva, showing the most stable surfaces compared to the initial and control sample, followed by Evetric.

### 3.2. Color Analysis

**Figure 4:**
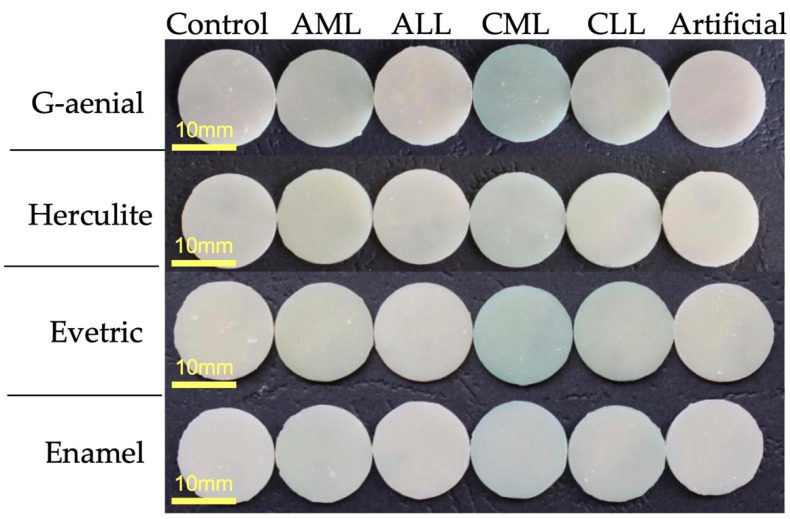
Color stability for dental composite.

All materials showed color changes during the 7 days of storage. Table 3 illustrates the reduction in the ΔE value depending on the saliva environment. A statistical evaluation of Anova one-way was performed between the same ΔE obtained at the same immersion time interval, but in different environments. For all dental composites investigated, no statistical significance was found between the immersion environment (Control, Initial, and Artificial Saliva) following the Tukey test. For the Evetric composite, the statistical differences between the environments are the most minimal (*p* = 2.05817 × 10^−4^), showing differences between the immersion environments and the Initial values, but not compared to the discs immersed in the Control environment. For the Enamel composite, (*p* = 4.549 × 10^−9^) most variations were obtained in the CML environment compared to other environments. The Herculite composite showed statistical differences in the AML, CLL, and AML environments, while the G-aenial composite showed differences between CML, CLL, and the rest of the environments (*p*=2.28931 × 10^−16^). For comparative studies of color changes, images captured digitally with a Zweiss Stemi 2000-C optical microscope were made for each material after immersion for 7 days in the six different saliva media (Figure 4) with a zoom range of 0.65X. According to the obtained images, a discoloration of the specimens visible to the naked eye for each dental material can be observed. Their behavior was similar: all four investigated materials have an insignificant difference in color change between the Control and Artificial environments, the most pronounced difference being in the CML environment. Enamel material is the most stable in terms of color change in all six types of saliva.

### 3.3. Saliva Elements Analysis

The results of the concentrations of the major elements in saliva samples are summarized in Table 4. The total concentrations of P, Na, and K showed wide ranges of variations, while the coefficient of variation in Fe, Cu, and Mg showed narrow variations between the salvias’ investigated. Comparisons between the results showed that the highest concentrations of elements are present in healthy saliva, and the lowest in artificial saliva and ALL saliva.

## 4. Discussion

Lymphoblastic leukemia deteriorates the surface of the Enamel composite, enhancing the erosive effects of saliva and modifying to a lesser extent the filler cluster shape and sizes. The most changed surface resulted for ALL (Figure 1Af), the roughness was almost double that of the untreated sample and significantly increased as compared to the healthy saliva. The effect of CLL is not as intense as observed for acute leukemia, but is significantly greater than for the one observed for the healthy state (Figure 1Ag). Lymphoblastic leukemia also deteriorated the Evetric composite surface in a manner caused by the excretion of some compounds. Its morphology was strongly modified by exposure to the ALL saliva (Figure 1Bf). An erosion of polymer between filler nano-particles occurs leading to the cluster’s diameter increasing to a range of 200–350 nm. This influences the surface roughness, which also increases (Table 2). The exposure to CLL saliva leads to a more significant increase in the clusters’ diameters, many of them being around 350–400 nm (some of them being over 500 nm) (Figure 1Bg). The roughness is not as high as the one observed for ALL, but it is significant higher than the healthy saliva. An explanation of the cluster increasing combined with slightly roughness decreasing could be the formation of some residual deposits from the saliva to the etched clusters in the Evetric composite. 

Acute lymphoblastic leukemia (ALL) has a strong influence on the surface of the G-aenial composite. Certain features of erosion occur deteriorating the fillers particles (Figure 1Cf); the submicron ones have a washed appearance and clusters are formed on the smaller filler particles, their diameter growing up to 90 nm. This could be explained by a complex interaction between the composite and some components excreted by the saliva. The measured roughness (Table 2) is comparable with the value obtained for healthy saliva, but the surface morphology is significant altered. The CLL has a stronger influence on the surface morphology (Figure 1Cg) with altered submicron filler particles in a range of 400–800 nm, and the nano-particles clusters growing up to 180–200 nm also becoming submicron constituents of the surface. Therefore, the surface roughness also increases to higher values than the ones observed for healthy saliva. ALL seems to modify some components of the saliva, which are able to significantly deteriorate the surface of the Herculite composite. ALL saliva promotes a significant dissolution of the initial feature of the samples and promotes nano-particle clusterization (Figure 1Df). The dissolution of nano-particles bonding into the polymer matrix leads to a relative increase in the diameter to about 90 nm. The partially de-binded nano-particles tend to reorganize into clusters of about 300 nm. This stage corresponds to a relative decrease in the surface roughness compared to the initial state. A more pronounced effect was observed for the CLL saliva where the roughness increased significantly due to the morphology distortion induced by severe erosion (Figure 1Dg). Two types of clusters were revealed: smaller in the range of 160–300 nm in diameter and large ones in the range of 600–900 nm. The morphological changes in the surface of the Herculite composite showed that CLL saliva acts as a strong erosive agent.

The changes in dental composite color are due to both exogenous factors (e.g., leukemia) [23] and endogenous factors (the characteristics of the composition of each resin) [24]. The discoloration of composites is closely related to the type of organic matrix used that influences the absorption [24,25]. Thus, dental composites with a higher filler ratio (80%) had a lower absorption capacity and, implicitly, a lower ΔE value (Evetric composite). The behavior of discoloration is also determined by the types of filling, and their size; Enamel and Herculite composites contain small filler particles (dimension 0.04–0.6 μ) that can be easily dispersed in the polymer matrix increasing the conversion degree and, for that reason, the values of ΔE are lower. The value of ΔE of the investigated materials increased in all six saliva environments; the greatest difference was seen between the initial values and the CLL and CML environments. However, all tested dental composites showed clinically acceptable color change values (ΔE < 3.3) in all four-leukemia salivas; CLL and CML showed large color differences for all composites. The Herculite composite showed the smallest difference (3.69 ± 0.79) in the CLL and CML environments.

The degradation and discoloration of dental materials may be also influenced by the chemical composition of leukemia saliva. Zinc is a stable mineral element that is involved in numerous reactions in the cellular metabolism, being responsible for both general and oral health [26]. In the current literature, it is stated that lower salivary flow determines higher concentrations of zinc [27,28]. It is well known that patients with leukemia develop oral mucositis with low salivary flow [4]. Surprisingly, the salivary concentration of zinc decreased considerably in the CLL and CML environments compared to the ALL and AML environments.

Human saliva has the ability to improve the process of remineralization of teeth by the presence of Ca and P ions. Some researchers have suggested that if there is calcium present, it should be at a high concentration to provide protection, with concentrations ranging from 60–100 mg/L Ca for healthy patients to 20–30 mg/L for those with periodontal disease [29,30]. The effect of periodontal disease showed the increase in Ca and Fe concentrations compared to the healthy group [31,32]. Another aspect that was stated was the significant increase in Cu and Na elements in the saliva of patients with severe periodontitis compared to healthy ones [33]. When it comes to our study, AML and ALL environments showed high concentrations of these elements. 

Among the inorganic components, Na and K are predominantly responsible for maintaining the osmolarity of saliva, while Ca, P, and Mg are responsible for maintaining the resistance of enamel to dental cavities [34,35,36]. Of the mineral elements targeted in the current study, Fe and Cu were significantly increased in the CML environment and Zn in the AML environment. These levels can lead to increased oxidative stress, which can cause tissue destruction [37]. Recently, it has been suggested that the Cu:Zn ratio could be a good indicator of the inflammatory condition [38]; so, in the CML and CLL environments, the inflammatory ratio was the highest. Low concentrations of Na, P, K, and Cu were seen in the ALL environment. There are very few studies on P and Mg concentrations in saliva; the limitations in their comparisons are difficult to be made. However, the mean values in Table 4 appear to indicate that the P concentrations are lower in all the six environments investigated. The Mg concentration in the control samples corresponded to those recently stated by Yu and collaborators [39]; the rest of the samples having lower values than those reported by Olmez and collaborators [40]. 

The behavior of RBCs in different salivas varies due to their characteristics and composition. The three-dimensional crosslinked structure of composites consisting of filler particles, polymeric matrix, and binders can change in different saliva media depending on their speed and degree of absorption. The character and the quantity of dilution plus the base of monomers mainly gives the absorption values of the composites, which, at a high degree of absorption, can modify their structure by breaking the polymeric bonds and releasing the filler particles. Another process may be due to reacted monomers from the polymer network, which influence the color stability, surface structure, and roughness of RBCs. This discoloration can be attributed to the degrees of hydrophilicity and absorption of fluids found in leukemia saliva. As seen in one of our previous studies, acute leukemia influenced more RBCs by changing their properties compared to chronic leukemia [41]. This behaviour was also found to be less in the AML, ALL, and CML saliva samples where the amount of residual UDMA is double that of TEGDMA.

Due to the lack of scientific literature on salivary leukemia and its influence on dental materials (e.g., dental composites), comparisons with other research could not be made. From our study, a few explanations may be drawn about the effects of salivary contamination on the longevity of restoration that may lead to sensitivity, discoloration of the teeth, and, finally, loss of restoration. One explanation could be the presence of xerostomia, which determines the alteration of the salivary composition. In regards to this, the accumulation of heavy dental plaque and calculus changes the composition of oral biofilm, which alters composition of human saliva in this frail patient. The observed erosive behavior of saliva from myeloid and lymphoblastic leukemia may also be caused by some components from chemotherapy, which are excreted into the saliva. Additionally, saliva of leukemia patients seems to attack the polymer matrix causing its partial dissolution, which induces modification of the filler particle disposal on the surface, discoloration of restorative materials, and, thus, the value and flow rate of oral flow that ensures patient health. 

Another important factor that should be taken into account is the chemotherapeutic regimen and its side effects on the oral mucosa and content of saliva. Degradation of RBCs is also influenced by oral microbes and by the mastication process [42]. In this case, saliva’s composition may contribute to the biodegradation process of the RBCs. This process was described by several authors where they stated that water molecules penetrate the polymer network, which allows the diffusion of unpolymerized monomers from the RBC network [43,44]. Another limitation of our research was the low number of participants; this fact might have influenced the statistical analysis. 

It is well studied in the literature and mentioned in oncological guidelines [14,15] that patients following chemotherapeutic regimens experience xerostomia, salivary gland dysfunctions, and oral mucositis, which change the oral health status of leukemia patients and increase the risk of dental decay. A clear conclusion about what kind of dental material should be use for decay is not stated.

In future studies, other RBCs should be analyzed according to the cycle of the chemotherapeutic regimen. Moreover, these RBCs should be studied in bone marrow transplant patients to conclude if they are safe to use in these frail patients. 

## 5. Conclusions

This in vitro pilot study showed that control saliva and artificial saliva have a mild erosive effect on the surface of dental composites, with minimal changes in morphology. Saliva from myeloid leukemia and lymphoblastic leukemia determined significant changes on surface morphology and roughness. Acute leukemia developed modifications on the surface roughness rather than on its morphology; meanwhile, in the chronic stage, surface morphology mostly seems to deteriorate. Results of this study should be interpreted with caution. More research in the assessment of dental composites in patients with leukemia are needed in order to have a clear conclusion. 

## Figures and Tables

**Table 1:** Composition of dental composites tested.

Material	Composition	Manufacturer
**G-aenial** Anterior A2,	- UDMA, Bis-GMA (37%) - pre-polymerized fillers containing silica (19-17 µ), pre-polymerized particles containing strontium (400 nm), lanthanoid fluoride (100 nm), silica (16 nm), fumed silica (63%)	GC corporation, Tokyo, Japan
**Herculite** XRV Ultra A2;	- Bis-GMA, TEGDMA (41%) - Al-B-Si glass, SiO_2_ (59% by volume, particles 0.6 µ)	Kerr, Bolzano, Italy
**Evetric** filling material A2,	- Dimethacrylates (19–20%) - barium glass, ytterbium trifluoride, mixed oxide, copolymers (80–81 wt.%; 55–57 vol.%, size 40–3000 nm)), additives, catalysts, stabilizers, pigments (80%)	Ivoclar Vivadent, Schaan, Liechtenstein
**Enamel** Plus HRi, UD2,	- UDMA, Bis-GMA, 1,4-butandiol-dimethacrylate (45%) - glass filler (0.7 µ), highly dispersed silicone dioxide (0.04 µ) (55%)	Micerium, Avegno, Italy

Bis-GMA: bisphenol A glycol dimethacrylate; TEGDMA: triethylene glycol dimethacrylate; UDM: urethane dimethacrylate.

**Table 2:** Mean values of the surface roughness.

Saliva	ENAMEL		EVETRIC		G-AENIAL		HERCULITE	
	Ra, nm	Rq, nm	Ra, nm	Rq, nm	Ra, nm	Rq, nm	Ra, nm	Rq, nm
Unexposed	5.28 ± 0.21	7.42 ± 0.83	6.82 ± 0.66	9.08 ± 1.07	6.38 ± 0.39	8.38 ± 0.28	7.37 ± 2.82	10.20 ± 3.18
Healthy	6.21 ± 2.23	8.42 ± 2.32	7.05 ± 2.15	8.85 ± 2.54	15.38 ± 2.23	19.40 ± 2.64	7.01 ± 2.67	9.08 ± 3.36
Artificial	6.76 ± 0.33	8.73 ± 0.51	10.0 ± 3.30	14.43 ± 6.35	15.68 ± 0.93	19.98 ± 1.02	5.92 ± 2.52	7.76 ± 3.22
AML	8.78 ± 3.55	11.4 ± 3.96	10.91 ± 2.35	15.6 ± 3.10	13.80 ± 3.55	17.58 ± 4.25	6.23 ± 3.59	9.36 ± 4.44
CML	6.24 ± 1.06	8.30 ± 1.43	8.97 ± 1.45	12.37 ± 2.58	13.53 ± 4.29	19.19 ± 6.61	5.91 ± 0.47	7.76 ± 0.84
ALL	10.32 ± 2.20	13.30 ± 2.57	9.02 ± 0.69	11.46 ± 1.05	11.17 ± 1.86	14.42 ± 2.33	6.15 ± 0.54	8.50 ± 1.15
CLL	7.56 ± 1.04	9.74 ± 1.53	8.66 ± 1.07	10.85 ± 1.05	16.42 ± 2.90	21.04 ± 4.03	17.28 ± 6.30	22.08 ± 9.40
*p* value	0.00369	6.19194 × 10^−4^	0.02203	0.01198	5.46568 × 10^−4^	0.00506	4.31649 × 10^−5^	3.99809 × 10^−4^

**Table 3:** Color changes in the dental composites’ using VITA EasyShade.

Dental Composite	ΔE ± SD Initial	ΔE ± SD after 7 Days of Immersion						*p* Value
		Control	Artificial	ALL	AML	CLL	CML	
G-aenial	23.15 ± 0.26	22.04 ± 1.04	20.74 ± 0.80	21.27 ± 1.06	20.68 ± 1.04	18.25 ± 1.81	15.28 ± 1.43	2.28931 × 10^−16^
Evetric	21.27 ± 0.55	18.98 ± 1.36	19.91 ± 1.68	19.67 ± 0.70	18.30 ± 0.85	17.29 ± 1.28	17.57 ± 2.48	2.05817 × 10^−4^
Herculite	22.55 ± 0.39	23.34 ± 1.45	21.63 ± 1.37	21.99 ± 1.30	19.46 ± 1.70	19.50 ± 1.04	18.85 ± 1.20	2.71886 × 10^−9^
Enamel	20.04 ± 0.05	18.97 ± 0.41	18.96 ± 0.64	18.65 ± 1.08	17.72 ± 0.96	16.88 ± 1.47	13.88 ± 1.19	4.549 × 10^−9^

**Table 4:** Concentrations of major elements in whole saliva.

Saliva	Zn (mg/L)	Ca (mg/L)	P (mg/L)	Fe (mg/L)	Cu (mg/L)	K (mg/L)	Mg (mg/L)	Na (mg/L)
Control	8.4246	47.8872	149.2041	3.7689	4.434	528.7545	7.5378	160.0541
Artificial	4.002524	19.54173	87.82008	4.002524	4.002524	375.5309	1.883541	152.3314
ALL	8.460541	28.80184	52.02333	3.060196	3.60023	140.949	5.220334	40.18635
AML	9.075908	32.34323	126.0726	2.970297	3.79538	492.5743	5.445545	66.3663
CLL	2.993056	21.74954	59.06297	2.993056	3.990741	171.2028	3.591667	41.15093
CML	3.506879	31.29215	72.56542	4.855678	5.125438	265.4438	4.855678	103.4985

## Data Availability

The data presented in this study are available on request from the corresponding authors.

## References

[1] Mester A., Irimie A.I., Tanase A., Tranca S., Campian R.S., Tomuleasa C., Dima D., Piciu A., Lucaciu O. (2020). Periodontal disease might be a risk factor for graft versus host disease. A systematic review. Crit. Rev. Oncol. Hematol..

[2] Murakami S., Mealey B.L., Mariotti A., Chapple I.L.C. (2018). Dental plaque-induced gingival conditions. J. Clin. Periodontol..

[3] Lynch M.A., Ship I.I. (1967). Initial oral manifestations of leukemia. J. Am. Dent. Assoc..

[4] Mester A., Irimie A., Oprita L., Dima D., Petrushev B., Lucaciu O., Campian R.-S., Tanase A. (2018). Oral manifestations in stem cell transplantation for acute myeloid leukemia. Med. Hypotheses.

[5] Angst P.D.M., Maier J., Dos Santos Nogueira R., Manso I.S., Tedesco T.K. (2020). Oral health status of patients with leukemia: A systematic review with meta-analysis. Arch. Oral Biol..

[6] Wang Y., Zeng X., Yang X., Que J., Du Q., Zhang Q., Zou J. (2021). Oral Health, Caries Risk Profiles, and Oral Microbiome of Pediatric Patients with Leukemia Submitted to Chemotherapy. Biomed Res. Int..

[7] Hong C.H.L., Napenas J.J., Hodgson B.D., Stokman M.A., Mathers-Stauffer V., Elting L.S., Spijkervet F.K.L., Brennan M.T. (2010). A systematic review of dental disease in patients undergoing cancer therapy. Support. Care Cancer.

[8] Hong C.H.L., Hu S., Haverman T., Stokman M., Napeñas J.J., Braber J.B., Gerber E., Geuke M., Vardas E., Waltimo T. (2018). A systematic review of dental disease management in cancer patients. Support. Care Cancer Off. J. Multinatl. Assoc. Support. Care Cancer.

[9] Hansen H.J., Estilo C., Owosho A., Solano A.K., Randazzo J., Huryn J., Yom S.K. (2021). Dental status and risk of odontogenic complication in patients undergoing hematopoietic stem cell transplant. Support. Care Cancer Off. J. Multinatl. Assoc. Support. Care Cancer.

[10] Kishimoto M., Akashi M., Tsuji K., Kusumoto J., Furudoi S., Shibuya Y., Inui Y., Yakushijin K., Kawamoto S., Okamura A. (2017). Intensity and duration of neutropenia relates to the development of oral mucositis but not odontogenic infection during chemotherapy for hematological malignancy. PLoS ONE.

[11] Tsuji K., Shibuya Y., Akashi M., Furudoi S., Yakushijin K., Kawamoto S., Okamura A., Matsuoka H., Komori T. (2015). Prospective study of dental intervention for hematopoietic malignancy. J. Dent. Res..

[12] Ertas E.T., Kurnaz F., Zorba Y.O., Kocyigit I., Sisman Y., Kaynar L., Sekerci A.E., Ertas H., Cetin M. (2014). Comparison of chemotherapy and hematopoietic stem cell transplantation pre and postterm DMFT scores: A preliminary study. Niger. J. Clin. Pract..

[13] Dobr T., Passweg J., Weber C., Tichelli A., Heim D., Meyer J., Gratwohl A., Waltimo T. (2007). Oral health risks associated with HLA-types of patients undergoing hematopoietic stem cell transplantation. Eur. J. Haematol..

[14] Elad S., Raber-Durlacher J.E., Brennan M.T., Saunders D.P., Mank A.P., Zadik Y., Quinn B., Epstein J.B., Blijlevens N.M.A., Waltimo T. (2015). Basic oral care for hematology-oncology patients and hematopoietic stem cell transplantation recipients: A position paper from the joint task force of the Multinational Association of Supportive Care in Cancer/International Society of Oral Oncology (MASCC). Support. Care Cancer.

[15] Elad S., Cheng K.K.F., Lalla R.V., Yarom N., Hong C., Logan R.M., Bowen J., Gibson R., Saunders D.P., Zadik Y. (2020). MASCC/ISOO clinical practice guidelines for the management of mucositis secondary to cancer therapy. Cancer.

[16] Münchow E.A., Ferreira A.C.A., Machado R.M.M., Ramos T.S., Rodrigues-Junior S.A., Zanchi C.H. (2014). Effect of acidic solutions on the surface degradation of a micro-hybrid composite resin. Braz. Dent. J..

[17] Tseng C.-C., Lin P.-Y., Kirankumar R., Chuang Z.-W., Wu I.-H., Hsieh S. (2021). Surface degradation effects of carbonated soft drink on a resin based dental compound. Heliyon.

[18] Szczesio-Wlodarczyk A., Sokolowski J., Kleczewska J., Bociong K. (2020). Ageing of Dental Composites Based on Methacrylate Resins-A Critical Review of the Causes and Method of Assessment. Polymers.

[19] Cheaib Z., Lussi A. (2011). Impact of acquired enamel pellicle modification on initial dental erosion. Caries Res..

[20] Berge T.L.L., Lygre G.B., Lie S.A., Lindh C.H., Björkman L. (2019). Bisphenol A in human saliva and urine before and after treatment with dental polymer-based restorative materials. Eur. J. Oral Sci..

[21] Agrawal V., Kapoor S. (2013). Color and shade management in esthetic dentistry. Univers. Res. J. Dent..

[22] Pröfrock D., Prange A. (2012). Inductively coupled plasma-mass spectrometry (ICP-MS) for quantitative analysis in environmental and life sciences: A review of challenges, solutions, and trends. Appl. Spectrosc..

[23] Ashok N.G., Jayalakshmi S. (2017). Factors that Influence the Color Stability of Composite Restorations Factors aFFecting color stability oF composite restorations. Int. J. Orofac. Biol..

[24] Shamszadeh S., Sheikh-Al-Eslamian S.M., Hasani E., Abrandabadi A.N., Panahandeh N. (2016). Color Stability of the Bulk-Fill Composite Resins with Different Thickness in Response to Coffee/Water Immersion. Int. J. Dent..

[25] Imamura S., Takahashi H., Hayakawa I., Loyaga-Rendon P.G., Minakuchi S. (2008). Effect of filler type and polishing on the discoloration of composite resin artificial teeth. Dent. Mater. J..

[26] Selow M.L.C., Vieira I., Tommasi M.H., Ritt A.C., Maistro A.E., Guerreiro A.L., Lange F., Souza J., Andrade J. (2008). Use of zinc in health from early childhood to late age. Alimentos e Nutrição Araraquara.

[27] Selow M.L.C., Lunelli F., Vieira I., Sotta M.D., Martins W.D.B., Ignacio S.A., Brancher J.A., De Oliveira Ribas M. (2016). Analysis of zinc concentration in the saliva of individuals at different age ranges. Rev. Odonto Cienc..

[28] Goto T., Komai M., Suzuki H., Furukawa Y. (2001). Long-term zinc deficiency decreases taste sensitivity in rats. J. Nutr..

[29] Romano F., Castiblanco A., Spadotto F., Di Scipio F., Malandrino M., Berta G.N., Aimetti M. (2020). ICP-Mass-Spectrometry Ionic Profile of Whole Saliva in Patients with Untreated and Treated Periodontitis. Biomedicines.

[30] Baumann T., Bereiter R., Lussi A., Carvalho T.S. (2017). The effect of different salivary calcium concentrations on the erosion protection conferred by the salivary pellicle. Sci. Rep..

[31] De Medeiros J.A.G., Zamboni C.B., Kovacs L., Lewgoy H.R. (2015). Investigation of Fe and Ca in non-stimulated human saliva using NAA. J. Phys. Conf. Ser..

[32] Monaci F., Bargagli E., Bravi F., Rottoli P. (2002). Concentrations of major elements and mercury in unstimulated human saliva. Biol. Trace Elem. Res..

[33] Dos Reis F.D., Pereira Júnior O.D.S., De Sousa R.A. (2020). Direct analysis of Na, K, Mg and Ca in human saliva and correlations with physiological conditions. Anal. Methods.

[34] Schützemberger M.E., Souza R.T., Petruccix R.E., Machado M.N., Papalexiou V., Brancher J.A. (2007). Análise bioquímica do fluido salivar de indivíduos portadores de doença periodontal (Biochemical analysis of saliva of subjects with periodontal disease). Endereço para correspondência. RSBO Revista Sul-Brasileira de Odontologia.

[35] Lewgoy H.R., Zamboni C.B., Metairon S., Medeiros I.M.M.A., De Medeiros J.A.G. (2013). Quantitative study of non-stimulated human whole saliva using NAA. J. Radioanal. Nucl. Chem..

[36] Zamboni C.B., Metairon S., Medeiros I.M.M.A., Lewgoy H.R. (2013). Investigation of saliva of patients with periodontal disease using NAA. AIP Conf. Proc..

[37] Burch R.E., Hahn H.K., Sullivan J.F. (1975). Newer aspects of the roles of zinc, manganese, and copper in human nutrition. Clin. Chem..

[38] Gaur S., Agnihotri R. (2017). Trace Mineral Micronutrients and Chronic Periodontitis—A Review. Biol. Trace Elem. Res..

[39] Yu B.S., Yuan Q.G., Nie L.H., Yao S.Z. (2001). Ion chromatographic determination of calcium and magnesium cations in human saliva and urine with a piezoelectric detector. J. Pharm. Biomed. Anal..

[40] Olmez I., Gulovali M.C., Gordon G.E., Henkin R.I. (1988). Trace elements in human parotid saliva. Biol. Trace Elem. Res..

[41] Mester A., Moldovan M., Cuc S., Tomuleasa C., Pasca S., Filip M., Piciu A., Onisor F. (2021). Characteristics of Dental Resin-Based Composites in Leukemia Saliva: An In Vitro Analysis. Biomedicines.

[42] Bettencourt A.F., Neves C.B., de Almeida M.S., Pinheiro L.M., e Oliveira S.A., Lopes L.P., Castro M.F. (2010). Biodegradation of acrylic based resins: A review. Dent. Mater..

[43] Kawahara T., Nomura Y., Tanaka N., Teshima W., Okazaki M., Shintani H. (2004). Leachability of plasticizer and residual monomer from commercial temporary restorative resins. J. Dent..

[44] Faltermeier A., Rosentritt M., Müssig D. (2007). Acrylic removable appliances: Comparative evaluation of different postpolymerization methods. Am. J. Orthod. Dentofac. Orthop..

